# Establishing a Multi-Country Sickle Cell Disease Registry in Africa: Ethical Considerations

**DOI:** 10.3389/fgene.2019.00943

**Published:** 2019-10-10

**Authors:** Nchangwi Syntia Munung, Victoria Nembaware, Jantina de Vries, Daima Bukini, Furahini Tluway, Marsha Treadwell, Raphael Zozimus Sangeda, Gaston Mazandu, Mario Jonas, Vivian Paintsil, Obiageli E. Nnodu, Emmanuel Balandya, Julie Makani, Ambroise Wonkam

**Affiliations:** ^1^Department of Medicine, University of Cape Town, Cape Town, South Africa; ^2^SickleInAfrica Data Coordinating Centre (SADaCC), University of Cape Town, Cape Town, South Africa; ^3^Division of Human Genetics, University of Cape Town, Cape Town, South Africa; ^4^Sickle Cell Programme, Muhimbili University of Health and Allied Sciences, Dar-es-Salaam, Tanzania; ^5^Department of Hematology/Oncology, UCSF Benioff Children’s Hospital Oakland, California, United States; ^6^Department of Pediatrics, UCSF School of Medicine, California, United States; ^7^Department of Pharmaceutical Microbiology, Muhimbili University of Health and Allied Sciences, Dar es Salaam, Tanzania; ^8^Department of Child Health, Komfo Anokye Teaching Hospital, Kumasi, Ghana; ^9^Center for Sickle Cell Disease Research and Training, University of Abuja, Abuja, Nigeria

**Keywords:** sickle cell disease, registries, SickleInAfrica, ELSI (ethical, legal, and social issues), Africa

## Abstract

Sickle cell disease (SCD) is one of the most prevalent genetic conditions in sub-Saharan Africa. It is a chronic, lifelong disease often characterized by severe pain. However, SCD has received little investment terms of health research, though there is currently a growing pool of SCD data from health and research facilities in different countries. To facilitate research on SCD in Africa, the SickleInAfrica consortium has established a SickleInAfrica registry. The registry will store a systematic collection of longitudinal data from persons with SCD across sub-Saharan Africa, and currently, participants are being enrolled in Ghana, Nigeria, and Tanzania. In establishing this registry, the SickleInAfrica consortium decided to actively identify and anticipate possible ethical issues that may arise in the development and management of the registry. This was motivated, in part, by the near absence of well documented ethical issues for registry research in Africa, more-so for registries enrolling participants across multiple countries and for a genetic condition. The consortium aims to establish standards for the equitable use of data stored in the registry. This paper presents a comprehensive report on the ethical considerations that came up in setting up a genetic disease registry across multiple African countries and how they were addressed by the SickleInAfrica consortium. Major issues included: active involvement of patients in the initiation and management of the registry; questions of assent and re-consent; the importance of ensuring that fears of exploitation are not replicated in African–African research collaborations; and the importance of public engagement in the management of registries. Drawing on this experience, SickleInAfrica plans to set up an ethics helpdesk for genetic disease registries and research in Africa.

## Introduction

Sickle cell disease (SCD) is a life-threatening monogenetic condition that affect millions of people globally (Ware et al., 2017). Persons with SCD often experience lifelong illness, chronic pain, fatigue, and a host of recurring and chronic health problems. SCD has been labelled a public health problem in sub-Saharan Africa (sSA) and under-5 morbidity and mortality rates for SCD in sSA are about 50–90% (Grosse et al., 2011; Wastnedge et al., 2018). Although there has been relatively little research and public health investment in SCD in most sSA countries, there is an increasing volume of clinical data on SCD in Africa (Makani et al., 2011; Wonkam et al., 2012; Diallo and Guindo, 2014). This data is, however, scattered in different public health facilities across Africa and this limits evidenced-based decision making on healthcare, research priorities and health policies for SCD in sSA. Given the contemporary push towards digital health and personalized medicine, an SCD registry would be a valuable resource for supporting research on SCD, as well as personalized or tailored healthcare for persons with SCD. Recognising the need and importance for an SCD registry, the Sickle Pan-African Research Consortium (SPARCo), in 2017, kick-started plans for a centralized SCD registry in sSA that will store clinical information of persons with SCD, including behavioral and demographic information, such as: age, gender, ethnicity, and family history. To achieve this objective, SPARCO collaborated with another African initiative, the SickleInAfrica data coordinating centre (SADaCC), to form the SickleInAfrica consortium, a consortium made up predominantly of African researchers and research institutions.

Enrollment to the registry is currently going on across multiple sites in three countries: Ghana, Nigeria, and Tanzania. It is hoped that the registry will facilitate research on SCD in Africa, including: clinical trials and implementation research for new interventions, genomics research, and behavioral studies. Furthermore, it is anticipated that the registry will facilitate improved health policies and care for SCD patients.

The SickleInAfrica registry will be jointly managed by SPARCo and SADaCC. SADaCC will serve as the data coordination hub and will help facilitate data collection and access procedures on behalf of the SickleInAfrica consortium. This includes: informatics and logistic support, addressing ELSIs, capacity building for data analysis at the different sites, and data governance. In this paper, we report on the ELSIs that the consortium had to address at the initiation and enrollment phase of the registry and how they were addressed by the consortium.

The SickleInAfrica registry provides an excellent case study to explore and discuss ELSIs in disease registries in Africa for a variety of reasons. Firstly, the SickleInAfrica registry is a multi-country registry involving different countries in Africa, each with different legal and/or institutional approaches to the recording, storing, and sharing and secondary use of health data. Secondly, many of the registry participants will be minors, which raises a number of ethical and social concerns that need to be appropriately addressed. Thirdly, being a registry for a genetic disease, it allows for a wider coverage of ELSIs compared to registries for diseases with no direct genetic linkage. Lastly, the SickleInAfrica consortium is predominantly an African–African (south–south) research collaboration and therefore presents an opportunity to critically highlight issues of equity and fairness in south–south research partnerships.

## Discussion

One of the key issues to focus on when initiating and running a disease-specific registry are the possible ethical legal and social issues (ELSIs) that may arise from the initiation and enrollment phase, up until when the data is shared for healthcare or research purposes (Davids et al., 2016). In the planning phase of the SickleInAfrica registry, the SickleInAfrica consortium intentionally opted to anticipate, identify and address possible ELSIs that may arise in the design and use of the Pan-African SCD registry. To achieve this, the consortium used a number of approaches. Firstly, SADaCC collected and reviewed informed consent forms that were being used for enrollment at the different SPARCo sites in Ghana, Nigeria, and Tanzania. SADaCC also analyzed the national health research guidelines for these three countries. The aim was to identify differences in data sharing regulation between the three participating countries. Thirdly, SickleInAfrica organized three consortium-wide meetings to discuss these ELSIs. Key ELSIs that were identified were related to: Informed consent, assent, and re-consenting; authorship and incentivizing data sharing; capacity building; intellectual property (IP) rights; benefits to study populations; and research priority setting. A brief description of these ELSIs and how the SickleInAfrica consortium opted to address them are presented below and in **Tables 1** and **2**.

**Table 1:** Review of national ethics guidelines and consent forms for participating SickleInAfrica sites.

Guideline/consent	Ghana	Nigeria	Tanzania	Comments and suggestions from ELSI core after review of consent forms and national regulation
National Guideline	Guideline under development	National Code of Health Research Ethics (2007) Policy Statement on Storage of Human Samples in Biobanks and Biorepositories in Nigeria (2013).	Guidelines of Ethics for Health Research in Tanzania (2009)	Guidelines for Nigeria and Tanzania support data sharing. The absence of a national guideline or regulation for Ghana will mean that approval by a REC is sufficient
Data sharing mentioned in consent forms	No	No	No	Include a statement that data will be shared with researchers other parts of the world
Storage period	No	No	No	Provide information on minimum period of storage or lifespan of the registry. For example, “your data will be stored for a minimum period of 10 years. If we are unable to maintain the registry, the match list may be destroyed”
Retrospective use of information	No	No	No	Add a statement that once enrolled to the registry, information related to SCD in their medical files will be extracted and included in the registry
Withdrawal	Yes	Yes	Yes	Additional statement required on what happens when a participant chooses to withdraw. For example: “If you decide to stop being part of the registry, your information that was already included in the registry will continue to be used for research purposes but no new information about you will be added to the registry”
Privacy and confidentiality	Not Specified	Not Specified	Not Specified	Additional statement that project staff will have access to anonymized and original data. Researchers will only have access to de-identified data
Who can consent	Parents (in the case of a minor).	Parent (assent and parental consent form available).	Parent (in the case of a minor)	Additional statement that when participant reach adulthood they will be given the opportunity to consent

**Table 2:** Recommendations for data governance in the SickleInAfrica registry.

Components of data sharing agreement for Tanzania	Tanzania Guideline	Recommendation by the SickleInAfrica consortium
Maximum Storage period	Not specified	A minimum period (10 years) needs to be included in the data sharing agreement.
Restriction to data sharing	Shared data can only be used for academic and research purposes. Transfer of data to third parties is not allowed, except for academic or research purposes, in which case, the recipient will have to secure the written consent of the provider.	Allow for maximum sharing of data for research that is linked to SCD including public health use other commercial activities that may support innovation, or the development of new interventions for SCD
Ownership of data	Provider institution will retain ownership of the data	Sites would decide on ownership rights and control of the data collected at the level of each country. SickleInAfrica will be custodian of aggregated data held in the central hub
Capacity building	The recipient of the data shall, in cooperation with the data provider, facilitate capacity building in data management and analysis in the provider’s institution.	Capacity building will be evidenced based. Sites will identify and prioritize their research capacity needs. SADaCC will support, facilitate and monitor capacity building activities at the different sites
Research publications	Recipient of data is expected to acknowledge the source of the data in all publications reporting use of the data. Recipient to make available to the data provider, at least 30 days before submission to a journal or presentation, manuscripts of publications emanating from use of the shared data.	SADaCC will develop, in consultation with SPARCo, an authorship policy taking cognisance of national regulation.
Intellectual property	The data provider shall be the sole owner of all rights and the title to the data transferred, including existing intellectual property rights.	Intellectual property rights may be retained by the SickleInAfrica while taking into consideration institutional policies
Benefit sharing	The Provider and Recipient shall discuss the sharing of benefits arising from use of the data in accordance with the contributions of the Parties	SickleInAfrica will maintain meaningful relationships with SCD patient groups, as key partners in the consortium. Benefit sharing arrangements would be discussed with all stakeholders on a case by case bases Other forms of benefit sharing include: capacity building, development of psychosocial and educational programs for SCD patients and their families.

## Informed Consent, Assent, and Re-Consenting

There have been suggestions that informed consent should be waived for registries (Tu et al., 2004). The main reason being that registries pose minimal risk and obtaining informed consent may be daunting (Davids et al., 2016). These arguments maybe stronger when the purpose of the registry is to collect data for policy or administrative purposes. However, in the case of registries that will be used for health research, informed consent is required, more so, consent for data sharing.

Analysis of informed consent forms used different SickleInAfrica sites showed that consent for data sharing was not being solicited at the time of enrollment (**Table 1**). Therefore data sharing, even other SickleInAfrica researchers will not be possible. Equally, although the SickleInAfrica registry will be storing data routinely collected as part of patient care, the data would be shared with researchers in different parts of the world. It was therefore important for consent for data sharing to be obtained. Also, given the risk of breach of privacy that comes with wide sharing of data, SickleInAfrica is of the opinion that it is legitimate for registry participants to have some level of control over how their data would be used for future studies. Therefore, the consortium opted for informed consent that allows participants to decide whether or not they would want to be contacted for future studies on SCDs.

During the consortium meetings, SickleInAfrica decided to suspend enrollment, revise the informed consent form to incorporate information on data sharing and to seek an amendment of ethics approval. In making this decision, SickleInAfrica also had to take into consideration the health research guidelines of the three countries given marked differences in country regulations for informed consent and data sharing in Africa (Munung et al., 2016; de Vries et al., 2017). In the three countries, consent for data sharing and unspecified health research were permissible in Nigeria and Ghana. In Tanzania, consent was only allowed for specified studies. The RECs of all three countries however approved data sharing following an amendment of the informed consent documents. This then required that already enrolled participants be re-consented.

A second ethical issue was assent for minors. SCD usually presents at infancy and therefore, a good number of persons to be enrolled in the registry will be minors (less than 18 years). Initially, only one of the three sites used an assent form for enrolling minors. In the two other sites, the approach was to get written parental consent and oral assent. Following the first consortium meeting, it was agreed that written assent forms should be used for children aged seven to seventeen. This was based on arguments that by age seven, children are able to understand certain aspects of research. This is however debatable (Wendler, 2006). The consortium therefore developed an assent form for the SickleInAfrica registry. Across the three countries, national RECs queried the comprehension of the assent forms by younger participants. Although SickleInAfrica finally developed an assent form that was acceptable by RECS in all three countries, a lot of time had gone into the process, thereby delaying enrollment. This demonstrates the need for model assent forms that could be used for enrollment in registries in Africa. While SickleInAfrica developed an assent form that was considered acceptable, a key point raised by the different RECs was the need to follow up on comprehension of the assent forms by minors as well as to develop tools that could be used with the assent forms. SickleInAfrica is in the process of developing educational materials that could be used during enrollment. It will also assess comprehension of assent forms as well as the supporting materials that would be used during enrollment.

A third issue related to informed consent was questions of re-consenting at the age of maturity, for persons who were enrollled as minors. This is important as the SickleInAfrica registry aims to collect longitudinal data from all registry participants. There was little regulatory guidance on this in the three participating countries. SickleInAfrica decided to get informed consent from all participants, once they reach maturity (18 years). At the time of re-consenting, participants will be asked if: 1) they would like to continue contributing data to the registry or 2) would want to discontinue participation. The choice to discontinue participation means that no new data about the patient will be added to the database and that already collected data will be deleted from the registry. However, information already in use in research projects will not be deleted.

The decision to re-consent was not without acknowledgment of possible logistical challenges to re-consenting research participants in African settings. These include: changes to residential and contact details; and that patients often do not use the same healthcare facility. Therefore, it will be difficult to keep track of registry participants, should they no longer be available at the contact details provided during enrollment. Equally, even when residential addresses remain the same, the lack of effective GIS systems in the participating African countries will make tracing difficult. Studies that document the processes of re-consenting in registries, including: the benefits, challenges, and possible solutions would be critical in addressing issues of re-consent in African research programs.

## Privacy and Confidentiality

Maintaining the privacy and confidentiality of persons enrollled in research projects or public health programs tends to be easier when there are no plans to share data outside of the specific project. However, in the case of registries where data will be accessed and used by different entities, either for research or public health purposes, questions of privacy and confidentiality become more complicated, even when data is anonymized or de-identified. The dominant idea in the consortium was to de-identify and encrypt data before it is shared. Whatever method of ensuring privacy, it is almost impossible to ascertain complete data protection in the digital age. Also, some longitudinal data, for example treatment outcomes, could be useful for individual patient care. Complete de-identification of data may therefore, present with a missed opportunity to provide improved personalized care for SCD patients. The consortium therefore decided to adopt solidarity based values when dealing with concerns of privacy. This requires ensuring maximum use of the data in ways that increase health benefits for SCD patients and their families. SickleInAfrica also agreed that in addition to solidarity, the principle of veracity is critical in guiding decisions around privacy and confidentiality. Therefore, all persons involved in patient enrollment, have the obligation to tell potential participants the truth related to data privacy, which is, that their data, even when de-identified, may be traceable to them. This statement was then included in the revised informed consent forms.

## Governance

Besides questions of informed consent, assent, privacy, and confidentiality, the other ELSIs were connected to secondary uses of data. These were mainly concerns of access to data, ownership/stewardship of data, acknowledgement of registry contributors, benefit sharing, intellectual property, capacity building, research priority setting, and authorship. Within the consortium, discussions around these ELSIs always seem to border on the democratization of data use and could therefore be broadly grouped as questions of governance.

### Data Sharing

The value of a registry lies more in how data will be used to address the burning health needs of the affected population. Therefore, a solidarity-based approach to the running and management of a registry might yield maximum value to persons with SCD and their families. This is especially relevant as registry participants stand to yield no direct benefit for contributing to the registry. SickleInAfrica researchers and patient support groups had concerns that their efforts in setting up the registry may not be recognized in the future. The researchers also expressed concerns that their use of the registry data is likely to be minimal given the limited research capacity in most African countries. It was therefore not surprising that one of the major data governance concerns centered on access to data in the registry and the conditions for data access. This included if the data should be limited to healthcare, research, and academic use; or if the data could be shared with pharmaceutical companies for the purposes of developing novel health interventions for SCD. While there was agreement that limiting data use to healthcare or research purposes only could stifle innovation and the rapid translation of research findings for the benefit of persons with SCDs, there were associated concerns that access by commercial entities would require that SickleInAfrica put additional measures for benefit sharing, patents, and intellectual property.

To address issues of data access, SickleInAfrica opted for managed access to data. This will be handled by a data access committee (DAC) set up by the consortium. The DAC will be made of members of the SickleInAfrica consortium; representatives of SCD support groups; and non-SickleInAfrica members. In deciding on access conditions, the DAC will prioritize projects that are done in collaboration with SickleInAfrica site investigators. This will not only ensure that the data is used to address the needs of SCD patients in Africa, but would also allow for capacity building and sustainability of research programs at the SickleInAfrica sites.

### Capacity Building

Generally, researchers tend to be reluctant to share data for fear that it could disadvantage the primary researchers (Walport and Brest, 2011; Carr and Littler, 2015). In global health research collaborations, data sharing has often been seen to pre-dispose African researchers to exploitation. This problem seems to also extend to south–south research collaborations. SickleInAfrica is an African–African research collaboration, but there are significant differences in research capacity at the different collaborating institutions. Discussions in the SickleInAfrica workshops consortium showed that participating sites with limited capacity for research and data analysis were reluctant to share their data. To address the problem, it was agreed that SADaCC would build capacity in data analysis at the different sites. This would hopefully empower site investigators to publish from the data even before the data is aggregated with those from other sites. SickleInAfrica also set up a skills working group that will map the available skills and capacity building needs at the different sites, thereby giving a chance to site PIs to determine first-hand what their needs are and how they could be supported by the consortium.

### Research Outputs, Authorship, and Acknowledgments

The SickleInAfrica registry will require substantial amount of efforts from researchers and healthcare providers at the different data collection sites. It was therefore questioned whether data collectors, producers, and curators will receive credits in research outputs, either as authors or as key contributors. The national guidelines for Tanzania requires that the primary data producers review, before publication, all scientific outputs emanating from data collected in Tanzania; and that the Tanzania investigator(s) are acknowledged in all publications. The guidelines in Nigeria and Ghana are silent on issues of authorship, therefore the consortium had to reach an agreement credits in scientific research outputs such as journal articles. An authorship policy was considered a more viable solution. Equally, it was agreed that in addition to the funders, key personnel at the sites should be acknowledged in publications arising from data in the SickleInAfrica registry.

Within the consortium, there was an expressed preference for site investigators to lead all publications arising from the use of the site data. This would ensure that the well-resourced institutions do not take advantage of the limited research capacity at the different sites, but rather support them to develop their own investigators and research interest. For projects involving a pool of data from the different sites, the lead authors would be decided by the research working group based on who initiated the study and/or demonstrated interest in exploring the specific question, while ensuring that there is an equal spread of responsibilities across the different sites.

A research output that received much deliberations but for which a definite policy could not be reached was the attribution of IP rights. There was a preference for IP rights to be allocated to the consortium rather than to researchers. However, the attribution of IP rights for innovations that may arise from use of the data in the SickleInAfrica registry will need to be aligned to institutional policies. The consortium will therefore need to consult with the IP offices of the different institutions before deciding on how to best articulate an IP policy for the consortium. This would include IPs for innovations that arise at the level of the consortium as well as from all other uses of data from the SickleInAfrica registry.

### Research Priority Setting

The operations and governance of the Sickle in Africa registry will be grounded in principles of solidarity and equity. The overarching goal of the registry is therefore not only to advance research on SCDs, but also to provide tailored and improved healthcare for persons with SCD. The principle of solidarity requires a collective commitment by various stakeholders to address the burning health problems of SCD patients in sSA. It was therefore not surprising that a key question in SickleInAfrica meetings was how the registry will address issues of morbidity and mortality that are linked to SCD in sSA.

To ensure that data from the Registry and the activities of the consortium align with health and research priorities for SCD in Africa, SickleInAfrica established a research working group. The main task of this group is to first and foremost identify research priorities for SCD in sSA. There search working group is made of investigators from the different sites and consists of healthcare workers who constantly provide care for persons with SCD. While the activities to identify and list research priorities where limited to researchers in the consortium, the plan is to extend it to SCD support groups and policy makers at both national and global level. The identified priorities will also serve as a guide for researchers interested in using data from the SickleInAfrica registry. This will ensure that researchers focuss more on the needs of persons with SCD, rather than the broader interest of persons/entities wishing to use data from the registry.

### Public Engagement and Patient Involvement in Registries

The usability and sustainability of the SickleInAfrica registry lies, to a large extent, on the ability SickleInAfrica to continuously update clinical information of participants. This will require maintaining long term relationships with registry participants. SickleInAfrica therefore decided to actively engage SCD patient groups in all four participating countries. So far, representatives from these patient groups have attended all, but one SickleInAfrica consortium meetings. During these initial stages, there has been little contributions from support groups, but a level of rapport has been established between SickleInAfrica and representatives of the SCD support groups. Support group leaders have also been engaged, as a stand-alone group in consortium meetings and have been involved in designing documents that would be used for enrollment and public engagement. There will however be need for a deeper and more systemic engagement of SCD patient support groups. An approach that is more appealing to SickleInAfrica is to have SCD support groups as key partners whereby these groups are involved in the design and initiation of the registry; the research planning phase; and in the governance of the registry. This may include involving them in community and engagement activities, decision making on use of data in the registry (for example in DACs); and in research priority setting.

Involving patient groups as partners in research is not without its own challenges (Solomon et al., 2017; Woodward et al., 2016). It requires giving them an active a voice in the governance of SickleInAfrica registry. While this may seem straightforward, there are very few examples of how this could possibly be achieved in resource limited settings. Our experience thus far is that SCD support groups would first need to have a clear understanding of the importance and benefits of an SCD registry. Together with the participating SCD support groups SickleInAfrica, is currently exploring ways by which SCD patient groups could be empowered to have an active presence in the governance of the SickleInAfrica registry, as well as in ensuring sustainability of the registry.

### Benefits to Study Communities

The operations of the SickleInAfrica registry will be espoused in the principle of solidarity. By extension, therefore, reciprocity would be another guiding principle of the consortium. Given the genetic nature of SCD, enrolling in an SCD registry that plans to share data poses significant privacy risk to patients and their families. There are also little direct benefits to them, except that they do so for the advancement of science. It is morally imperative that SickleInAfrica also identify ways by which the SCD patient community may benefit from the operations of the registry.

SickleInAfrica struggled to articulate possible direct benefits to registry participants or their communities. Initial discussions with representatives of SCD support groups in the four countries suggest that SCD patients and their families may benefit from psycho-social support as well as educational programs on SCD. It was observed that healthcare workers currently have limited understanding on how to manage persons with SCDs and this hampers proper care for SCD patient especially when they are experiencing a health crisis. To address this problem, SickleInAfrica is in the process of setting up programs which aim at understanding the psychosocial issues experienced by SCD patients and their families and how they could be appropriately supported. In addition, SickleInAfrica is also developing educational resources that would empower SCD patient groups and healthcare workers to better support persons with SCD and/or their families. While these may not be the primary objective of the SickleInAfrica registry, it would ensure that SCD patients and their families, feel supported while contributing to the common good. The educational programs and resources developed as part of these activities will be made widely available to other interested SCD programs. Also, given the legitimate need for patients to be aware of how their data is being used, SickleInAfrica will maintain meaningful relationships with SCD patient groups, as key partners in the consortium, and therefore will likely adopt a power-sharing model for governance (Winickoff, 2008), that will allow study communities (SCD support groups) to have a say in the benefit sharing approach as and when necessary.

## Conclusion

There is little existing ELSI support for institutions that plan to establish multi-country health registries in Africa. Drawing on our experience of setting up a multi-country African SCD registry, we report on ELSIs that are likely to emerge when setting up disease registries in Africa. We also highlight (**Table 2**) how the SickleInAfrica consortium managed or is currently navigating through these ELSIs, including but not limited to: developing equitable policies for data sharing, having open discussions with all stakeholders; and developing an active patient and public engagement model for disease specific registries in Africa. SickleInAfrica will also create an ELSI helpdesk for genetic disease registries in Africa. Through this helpdesk, the consortium will make publicly available the ELSI resources developed by the consortium as well as provide support to similar initiatives.

Discussions on ELSIs in biobanking in Africa (Tindana et al., 2014; de Vries et al., 2017; Munung et al., 2017) provide a good base for thinking about ethical issues that may arise in setting up and running multi-country genetic disease registries in Africa. However, much still needs to be invested in developing ELSI support frameworks for disease specific registries in Africa. SickleInAfrica is currently developing a number of guidelines that could be used as models by similar initiatives in Africa. Of particular importance is the need to identify an adequate age group for assent in genetic disease registries, as well as to develop assent forms that are comprehensible to the target age group. There is also need for empirical studies that explore: the role of assent in health research in Africa; and comprehension of research concepts by children enrolled in genetic studies.

The near absence of regulation for data sharing in most African countries (de Vries et al., 2017), is a challenge for establishing registries in Africa. Yet, it is also a good opportunity to leverage on in harmonizing data sharing regulation such that it supports the interoperability of multi-country registries across the continent. The focus of such regulation should not be on minimizing harm that is unpredictable, rather, it should be about maximizing data use and increasing benefits for study populations. In the absence of harmonized regulation, an alternative will be to develop a governance model that are informed both by the principles of solidarity, reciprocity, and equity (Sethi, 2018).

In setting up the SickleInAfrica registry, it became clear that there are also strong concerns of exploitation and inequitable collaborations in African–African research collaborations. While there are efforts to address concerns of inequities in collaborations between LMICs and HICs, these discussions are yet to be extended to collaborations between African research institutions. SickleInAfrica is one of few examples of an African–African research collaboration and has a chance to showcase how justice and fairness could be advanced in African–African research collaborations. It is also an opportunity to unpack concerns that African researchers may have with respect to collaborating with other African researchers; and to design a model for south–south research collaborations.

Lastly is the need for stakeholder engagement. The SickleInAfrica registry will benefit from active involvement of SCD patient groups. The Genetic Alliance as well as some rare disease registries have made suggestions on how patient support groups could be involved in registry development and governance, and the benefits thereof (Terry et al., 2011; Workman, 2013). The consortium is therefore making efforts to consider the experiences, perspectives, and priorities of SCD patients in its governance strategy. This is because it is in the legitimate interest of registry participants to have some degree of control, as well as awareness, of how their data is being used.

## Data Availability Statement

All datasets generated for this study are included in the manuscript/Supplementary Files.

## Author Contributions

AW and JM conceived and supervised the work reported in this paper. NM, VN, and DB analyzed the informed consent forms and national regulatory guidelines. All authors contributed to: the ethics discussions in consortium meetings which involved highlighting the ethical issues that are pertinent to SickleInAfrica. The paper was first drafted by NM, VN, and JV. All authors read and commented on drafts of the manuscript. The final manuscript was approved by all authors.

## Funding

This work was supported by the National Heart, Lung, and Blood Institute of the National Institutes of Health (Award Number U24HL135600). NM and JV received support from the Stigma in African Genomics H3Africa project (Award number 1U01HG008226-01) administered by the National Human Genome Research Institute. DB, FT, VP, RS, ON, EB, and JM are funded by the National Heart, Lung and Blood Institute of the National Institutes of Health (Award Number 1U24HL135881). The content of this paper is solely the responsibility of the authors and does not necessarily represent the official views of the funders.

## Conflict of Interest

The authors declare that the research was conducted in the absence of any commercial or financial relationships that could be construed as a potential conflict of interest.
